# Analysis of predictors after partial splenic embolization for thrombocytopenia with liver cirrhosis

**DOI:** 10.1097/MD.0000000000030985

**Published:** 2022-10-07

**Authors:** Toru Ishikawa, Kazuki Ohashi, Erina Kodama, Takamasa Kobayashi, Motoi Azumi, Yujiro Nozawa, Akito Iwanaga, Tomoe Sano, Terasu Honma

**Affiliations:** a Department of Gastroenterology, Saiseikai Niigata Hospital, Niigata, Japan; b Faculty of Health Sciences, Hokkaido University, Sapporo, Japan.

**Keywords:** IL-6, PA-IgG, partial splenic embolization, splenic infarction rate, thrombocytopenia, thrombopoietin

## Abstract

Blood transfusion, splenectomy, and partial splenic embolization (PSE) are generally performed for thrombocytopenia in patients with cirrhosis. Recently, thrombopoietin (TPO) agonists have become available, and investigations of patients who would benefit from them are necessary. Therefore, it is important to understand the fluctuations in cytokine levels associated with PSE. Therefore, fluctuations in platelet-associated immunoglobulin G (PAIgG), interleukin 6 (IL-6), and TPO levels with PSE were analyzed in this study. The study included 110 patients with liver cirrhosis and thrombocytopenia, with the aim of improving platelet counts. Fluctuations in PAIgG, IL-6, and TPO levels were investigated. The average splenic embolization ratio was 58.0% in patients with PSE. The platelet count rose significantly from 6.95 [5.40, 8.60] × 10^4^/mL to 14.05 [10.43, 18.05] × 10^4^/mL (*P* < .01), IL-6 rose significantly from 3.56 [2.53, 7.33] pg/mL to 18.90 [9.17, 32.95] pg/mL (*P* < .01), TPO rose significantly from 0.82 [0.52, 1.21] fmol/mL to 1.58 [0.97, 2.26] fmol/mL (*P* < .01), and PAIgG decreased significantly from 64.20 [38.33, 118.75] ng/10^7^ cells to 37.50 [22.25, 70.00] ng/10^7^ cells (*P* < .01). On multivariate analysis of factors related to the rate of platelet increase with PSE, primary biliary cholangitis (B = 0.475, *P* < .01), splenic embolization ratio (B = 0.75, *P* < .01), IL-6 change ratio (B = 0.019, *P* < .01), and PAIgG change ratio (B = −0.325, *P* < .01) were significant. When attempting to improve thrombocytopenia with PSE, adequate splenic embolization needs to be obtained together with improvements in IL-6, PAIgG, and TPO levels. With unsatisfactory improvement in thrombocytopenia, TPO agonist administration was considered.

## 1. Introduction

Thrombopoietin (TPO) is a major hematopoietic factor that facilitates platelet production. Over 27 years have passed since several groups successfully cloned TPO, each with its own method, around the same time in 1994.^[1–3]^ Much basic research have been conducted since TPO was first cloned, and various findings have been obtained, including the action of TPO in the hematopoietic system and its molecular structure, production-regulating mechanism, and signaling mechanism. Research on TPO is advancing, and TPO agonists have been shown to be useful even for thrombocytopenia in chronic liver disease.^[4]^

It has also been reported that anti-platelet antibodies (platelet-associated immunoglobulin G [PAIgG]) present in the blood in chronic liver disease are a factor in thrombocytopenia.^[5,6]^ In particular, PAIgG was seen in more than 80% of patients with chronic hepatitis C, and the antibody titer was reported to have a good inverse correlation with the platelet count.^[7]^

These findings suggest that immunological mechanisms can induce thrombocytopenia. Moreover, in chronic liver disease, splenomegaly is observed as the condition progresses, and there is a correlation between the size of the spleen and the degree of thrombocytopenia due to accelerated platelet destruction.^[8]^

Interleukin-6 (IL-6) is a cytokine for which gene cloning has been performed as a B cell differentiation factor. However, it has been reported, mainly from in vitro investigations, that it also acts on cells of the hematopoietic system, such as megakaryocytes and hematopoietic stem cells. It is an important cytokine involved in thrombocytosis, and together with TPO, it has attracted attention as a cytokine associated with platelet increase.^[9]^

The mechanism of thrombocytopenia in chronic liver disease involves a primary factor for hypersplenism. Therefore, in recent years, partial splenic embolization (PSE) and splenectomy have been actively performed for thrombocytopenia associated with hypersplenism. However, these procedures are invasive, and, currently, TPO receptor agonists are promising, even when used clinically.

Knowledge of the changes in cytokines with PSE for thrombocytopenia in chronic liver disease associated with splenomegaly may be useful in determining the indications for TPO receptor agonists. Therefore, fluctuations in PAIgG, IL-6, and TPO with PSE for thrombocytopenia in patients with chronic liver disease due to hypersplenism were investigated.

## 2. Methods

Twenty hundred eight patients with liver cirrhosis and thrombocytopenia underwent PSE for hypersplenism between November 2007 and September 2021, and who did not meet any of the following exclusion criteria were included in the study. Patients with complications from hepatocellular carcinoma (HCC), those with missing data, and those in whom interventions such as platelet transfusion were already being used were excluded from the study. The subjects were 110 patients with liver cirrhosis and thrombocytopenia, with the aim of improving platelet counts were analyzed.

PSE was performed according to the Takatsuka method, in which a catheter is inserted near the splenic hilum, and splenic arteriography is performed, after which each branch is identified from an arterial map within the spleen, microcatheters are selectively inserted into the vessels, and embolization is performed using a coil and a 2-mm^2^ gelatin sponge soaked in 20 g of gentamicin.^[10]^ An intravenous drip of antibiotics was administered given via a peripheral vein for three days after PSE, and an oral non-steroidal anti-inflammatory drug was taken as needed when the patient’s temperature rose to over 38°C. The splenic embolization ratio from PSE was measured using angio-CT immediately after PSE. The trends in platelet count, IL-6 value, PAIgG, and TPO values were investigated before PSE and two weeks after PSE.

### 2.1. Ethics statement

The study was approved by the Institutional Review Board of Saiseikai Niigata Hospital (approval number: E18-18) and performed in accordance with the principles of the Declaration of Helsinki (as revised in 2013). Written informed consent was obtained from all patients.

### 2.2. Statistical analysis

Continuous variables are expressed as medians and quartiles (quartile 1; quartile 3). Nominal variables were reported as frequencies and percentages. Clinical characteristics were compared between the pre- and post-PSE groups using the Wilcoxon signed-rank test. The change ratio of platelets (post-platelet counts—pre-platelet counts/pre platelet counts), which was an objective variable, was transformed into a natural logarithm after adding 1. Univariate and multiple linear regression analyses were performed to explore the factors related to an increased platelet count, such as age, sex, etiology, presence of HCC, splenic embolization ratio, IL-6 change ratio, PAIgG change ratio, and TPO change ratio. All analyses were performed using EZR (Saitama Medical Center, Jichi Medical University), which is based on R (The R Foundation for Statistical Computing). Statistical significance was set at *P* < .05 was considered significant.^[11]^

## 3. Results

The median age of the participants was 63 [56.00, 68.75] years, and they included 65 men (59.1%) and 45 women (40.9%). The etiology was hepatitis B virus in 10 patients (9.1%), hepatitis C virus (HCV) in 44 (40.0%), alcohol in 34 (30.9%), nonalcoholic steatohepatitis (NASH) in 11 (10.0%), primary biliary cholangitis (PBC) in 6 (5.5%), and autoimmune hepatitis in 5 (4.5%). Concurrent HCC was present in 29 patients (26.4%). The splenic volume was 408.39 [247.44, 579.59] cm^3^, platelet count was 6.95 [5.40, 8.60] × 10^4^/mL, IL-6 was 3.56 [2.53, 7.33] pg/mL, PAIgG was 64.20 [38.33, 118.75] ng/10^7^ cells, and TPO was 0.82 [0.52, 1.21] pg/mL (Table 1).

**Table 1:** Patients’ baseline characteristics.

Variables	Overall (n = 110)
Age, yr	63.00 [56.00, 68.75]
Sex, male [n (%)]	65 (59.1)
Etiology	
HBV	10 (9.1)
HCV	44 (40.0)
Alcohol	34 (30.9)
NASH	11 (10.0)
PBC	6 (5.5)
AIH	5 (4.5)
HCC, present	29 (26.4)
Spleen size (cm^3^)	408.39 [247.44, 579.59]
Platelets (×10^4^/mL)	6.95 [5.40, 8.60]
IL-6 (pg/mL)	3.56 [2.53, 7.33]
PAIgG (ng/10^7^ cells)	64.20 [38.33, 118.75]
Thrombopoietin (fmol/mL)	0.82 [0.52, 1.21]

Values are presented as medians [interquartile range].

AIH = autoimmune hepatitis, alcohol = alcoholic hepatitis, HBV = hepatitis B virus, HCC = hepatocellular carcinoma, HCV = hepatitis C virus, IL-6 = interleukin 6, NASH = nonalcoholic steatohepatitis, PAIgG = platelet-associated IgG, PBC = primary biliary cholangitis.

The average splenic embolization ratio was achieved in 58.0% of cases, and the platelet count rose significantly from 6.95 [5.40, 8.60] × 10^4^/mL to 14.05 [10.43, 18.05] × 10^4^/mL (*P* < .01), IL-6 rose significantly from 3.56 [2.53, 7.33] pg/mL to 18.90 [9.17, 32.95] pg/mL (*P* < .01), TPO rose significantly from 0.82 [0.52, 1.21] fmol/mL to 1.58 [0.97, 2.26] fmol/mL (*P* < .01), and PAIgG decreased significantly from 64.20 [38.33, 118.75] ng/10^7^ cells to 37.50 [22.25, 70.00] ng/10^7^ cells (*P* < .01) (Table 2).

**Table 2:** Comparison of clinical characteristics between before and after partial splenic embolization.

Variables	Pre PSE	Post PSE	Change rate (post-pre/post)
Platelets (×10^4^/mL)*	6.95 [5.40, 8.60]	14.05 [10.43, 18.05]	1.00 [0.59, 1.61]
IL-6 (pg/mL)*	3.56 [2.53, 7.33]	18.90 [9.17, 32.95]	3.14 [1.05, 6.31]
PAIgG (ng/10^7^ cells)*	64.20 [38.33, 118.75]	37.50 [22.25, 70.00]	−0.35 [−0.56, −0.19]
Thrombopoietin (fmol/mL)*	0.82 [0.52, 1.21]	1.58 [0.97, 2.26]	0.62 [0.25, 1.42]
Splenic embolization ratio		0.58 [0.49, 0.70]	

Values are presented as medians [interquartile range].

IL-6 = interleukin 6, PAIgG = platelet-associated IgG, PSE = partial splenic embolization.

* *P* < .01 using the Wilcoxon signed-rank test.

**Figure 1.**
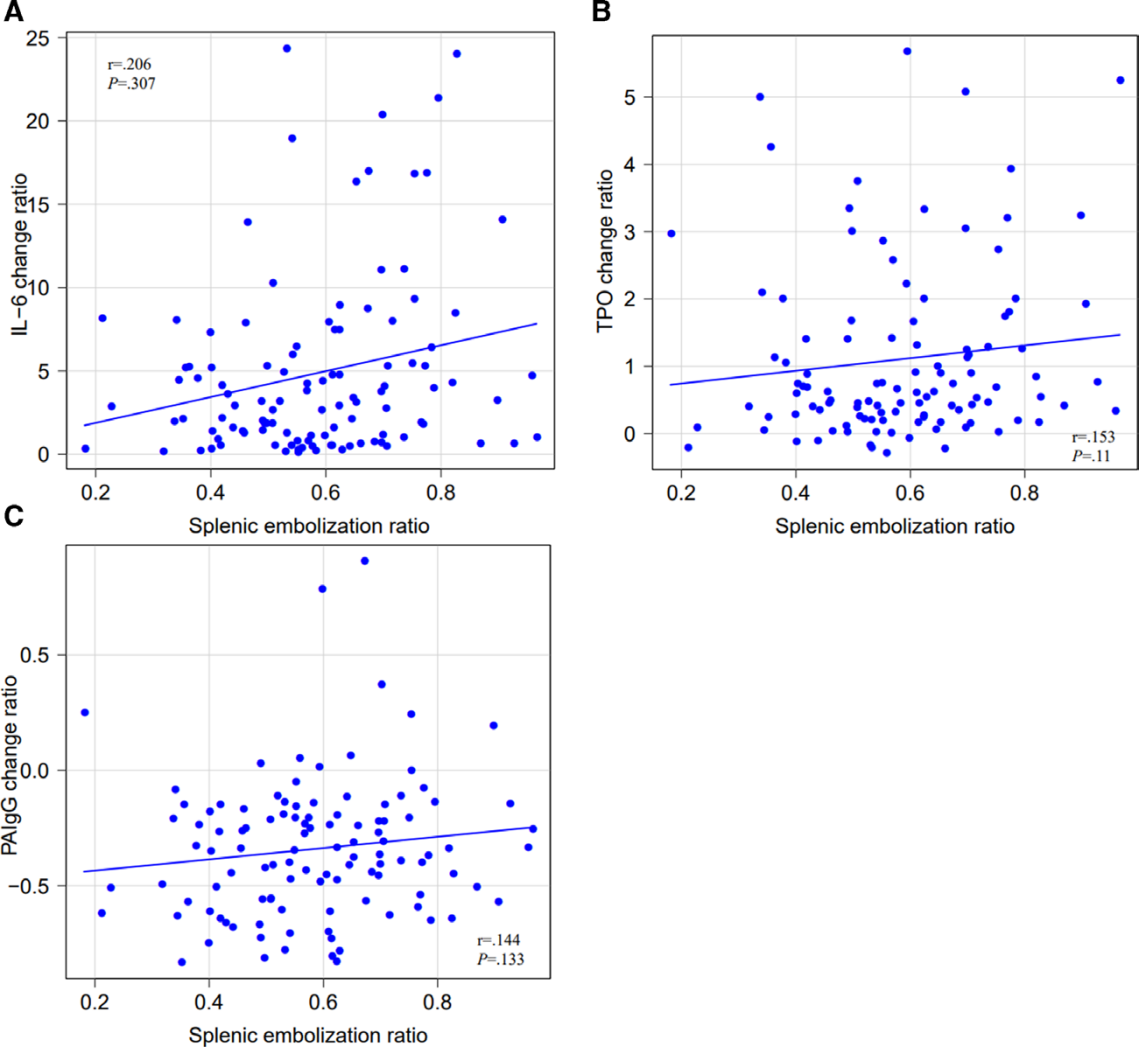
(A) Correlation between the IL-6 change ratio and the splenic embolization ratio (Spearman’s rank correlation coefficient). *r* = 0.206, *P* = .0307. (B) Correlation between the TPO change ratio and the splenic embolization ratio (Spearman’s rank correlation coefficient). *r* = 0.153, *P* = .11. (C) Correlation between the PAIgG change ratio and the splenic embolization ratio (Spearman’s rank correlation coefficient). *r* = 0.144 *P* = .133. IL-6 = interleukin 6, PAIgG = platelet-associated immunoglobulin G, TPO = thrombopoietin.

The correlation between the splenic embolization ratio and the rates of change in IL-6, PAIgG, and TPO was investigated, and a positive correlation was observed only with the IL-6 change ratio (Fig. 1).

Factors related to the rate of increase in platelets due to PSE were investigated by univariate analysis, and the following were identified: the etiology of liver disease (HCV, PBC, and NASH), splenic embolization ratio, IL-6 change ratio, and PAIgG change ratio. On multivariate analysis, PBC (B = 0.475, *P* < .01), splenic embolization ratio (B = 0.75, *P* < .01), IL-6 change ratio (B = 0.019, *P* < .01), and PAIgG change ratio (B = −0.325, *P* < .01) were significant (Table 3). No serious complications occurred after PSE.

**Table 3:** Univariate and multivariate analyses of factors associated with an increase in platelets.

Variables	Univariate			Multivariate		
	Coefficient	95% CI	*P* value	B	95% CI	*P* value
Age, yr	0.003	−0.005 to 0.011	.44	0.001	−0.006 to 0.009	.71
Sex, male	−0.005	−0.163 to 0.152	.95	0.092	−0.061 to 0.245	.23
Etiology						
HBV	0.038	−0.231 to 0.307	.78			
HCV	−0.258	−0.408 to −0.108	<.01	−0.127	−0.273 to 0.200	.09
Alcohol	0.004	−0.163 to 0.172	.96			
NASH	0.262	0.009–0.515	.04	0.153	−0.089 to 0.395	.21
PBC	0.422	0.092–0.754	.01	0.475	0.155–0.795	<.01
AIH	0.288	−0.079 to 0.655	.12			
HCC, present	−0.039	−0.215 to 0.136	.66			
Splenic embolization ratio	0.851	0.399–1.303	<.01	0.75	0.329–1.170	<.01
IL-6 change rate	0.024	0.010–0.037	<.01	0.019	0.006–0.031	<.01
PAIgG change rate	−0.298	−0.552 to 0.045	.02	−0.325	−0.545 to −0.106	<.01
Thrombopoietin change rate	0.032	−0.028 to 0.091	.29			

*R*-squared = 0.367, adjusted *R*-squared = 0.317, *F*-statistic = 7.312, *P* < .01, sample size of 110.

AIH = autoimmune hepatitis, alcohol = alcoholic hepatitis, B = partial regression coefficient, CI = confidence interval, HBV = hepatitis B virus, HCC = hepatocellular carcinoma, HCV = hepatitis C virus, IL-6 = interleukin 6, NASH = nonalcoholic steatohepatitis, PAIgG = platelet-associated IgG, PBC = primary biliary cholangitis.

## 4. Discussion

In patients with liver cirrhosis, splenomegaly (hypersplenism) occurs together with the progression of liver fibrosis, and the platelet count decreases. To date, the mechanism of thrombocytopenia in chronic liver disease has been considered to be hypersplenism.

Anti-platelet antibodies (PAIgG) that are present in the blood, even in conditions other than hypersplenism, are a factor in thrombocytopenia.^[7]^ Recently, Splenectomy and PSE for hypersplenism in patients with chronic liver disease and thrombocytopenia have attracted attention. Splenectomy has been reported as a treatment for Banti’s disease^[12]^ and led to a focus on the spleen as the origin of portal hypertension. Splenic artery embolization was first reported by Maddison in 1973,^[13]^ but its indications were initially limited to severe complications, such as splenic abscess and septic pneumonia. However, PSE that restricted the range of embolization was reported by Spigos et al in 1979,^[14]^ and came to be widely used clinically after its safety was improved. PSE, like splenectomy, PSE improves hepatic function, hypersplenism, portal hypertension, and gastroesophageal varices, as well as increases the platelet count,^[15]^ and is also reported to be effective in IFN therapy for hepatitis C with thrombocytopenia^[16]^ and in the treatment of HCC with thrombocytopenia.^[17,18]^

PSE is useful for increasing platelets; however, fluctuations in cytokines before and after PSE for liver cirrhosis with thrombocytopenia and hypersplenism have not been adequately investigated. In addition, although there are many TPO agonists, it is important to know how cytokines fluctuate with PSE when deciding which one is suitable. In this study, the fluctuations in TPO, IL-6, and PAIgG levels before and after PSE for thrombocytopenia were investigated in patients with liver cirrhosis and hypersplenism.

When factors related to the change in platelet ratio from PSE were investigated by univariate analysis, four items were identified: etiology (HCV, PBC, and NASH), splenic embolization ratio, IL-6 change ratio, and PAIgG change ratio. In multivariate analysis, platelet ratio (amount of change/pre-PSE platelet count) increased by approximately48% when the etiology was PBC.

Spleen volume and PAIgG have been suggested to be involved in CLD thrombocytopenia,^[6]^ but in the present study, it is unclear whether IL-6 also plays a role.

TPO, a factor in platelet production, was identified and cloned in 1994. The liver, in particular hepatocytes, is the main organ that produces TPO. The possibility of platelet production failure due to decreased expression of TPO from hepatocytes has been reported as one factor in the thrombocytopenia of cirrhosis.^[19]^ This phenomenon is something that does not occur in acute hepatitis, fulminant hepatitis, and other conditions.^[20]^ However, there are many reports that TPO is constant or slightly decreased in the blood of cirrhosis patients. One reason for this is that expression of the TPO receptor is related to the degree of cirrhosis progression.^[21]^

In the present study, there was a significant increase in TPO levels with PSE. A decreasing trend in PAIgG levels was also observed. In contrast, IL-6 levels increased significantly. In chronic liver disease, platelet count decreases with progression from chronic hepatitis to cirrhosis. The mechanism of thrombocytopenia in liver cirrhosis is considered to be hypersplenism, but an additional main cause is failure of the liver, the main production organ, to produce TPO.

Therefore, it is possible that patients whose platelets do not improve with PSE include some who fail to produce TPO from the liver even with PSE; therefore, a TPO agonist may be needed in patients in whom TPO it is not sufficient, although it tends to increase somewhat.

Shimodaira et al^[22,23]^ reported that, with splenectomy in cirrhosis patients, platelets counts increase, but the TPO value remained unchanged.

Of the TPO fluctuations seen in various conditions in which thrombocytopenia occurs, the fluctuations with ITP are reported to differ from those with aplastic anemia or post-chemotherapy bone marrow suppression.^[24]^ That words, in conditions such as aplastic anemia or post-chemotherapy bone marrow suppression where blood TPO rises, TPO is replenished, and the amplification of production signals with the addition of a TPO analog or via TPO receptors is thought to be difficult to obtain. Meanwhile, with ITP or PSE for cirrhosis in the present study, blood TPO levels did not increase much, and therefore, an effect of TPO receptor agonists was expected. Hence, as with all TPO agonists, there is a potential risk of thromboembolism, which must be monitored. The risk of portal vein thrombosis and hepatotoxicity often occurrs in patients with chronic liver disease.^[25]^ Furthermore, re-administration may excessively increase the platelet count, thus increasing the risk of thromboembolism.^[26]^

This study had several limitations. First, the sample size is small. Confirmation of these findings will require larger, prospective, clinical trials. Second, it was unclear whether other cytokines showed similar results. Third, the data were from a single center, and selection bias could not be ruled out.

In conclusion, this study suggests that one possible factor in the increase in platelets with PSE involves the increase in IL-6 more than the elevation in TPO itself or PAIgG immunological mechanisms. It has also been reported that platelets are maintained for a long period of 6 months or even 4 years if PSE is effective.^[27,28]^

However, in patients whose platelets do not increase with PSE, there is thought to be another route to increase platelets with TPO agonists, which appears promising for liver cirrhosis patients with thrombocytopenia.

## Acknowledgements

We would like to thank FORTE Science Communications (www.forte-science.co.jp) for the English language editing.

## Author contributions

**Conceptualization:** Toru Ishikawa.

**Data curation:** Toru Ishikawa, Kazuki Ohashi, Erina Kodama, Takamasa Kobayashi, Motoi Azumi, Yujiro Nozawa, Akito Iwanaga, Tomoe Sano.

**Formal analysis:** Toru Ishikawa.

**Supervision:** Toru Ishikawa.

**Validation:** Toru Ishikawa, Kazuki Ohashi.

**Writing – original draft:** Toru Ishikawa.

**Writing – review & editing:** Toru Ishikawa, Kazuki Ohashi, Erina Kodama, Takamasa Kobayashi, Motoi Azumi, Yujiro Nozawa, Akito Iwanaga, Tomoe Sano.
